# High blood sugar levels significantly impact the prognosis of colorectal cancer patients through down-regulation of microRNA-16 by targeting *Myb* and *VEGFR2*

**DOI:** 10.18632/oncotarget.7719

**Published:** 2016-02-25

**Authors:** I-Ping Yang, Hsiang-Lin Tsai, Ching-Wen Huang, Chien-Yu Lu, Zhi-Feng Miao, Se-Fen Chang, Suh-Hang Hank Juo, Jaw-Yuan Wang

**Affiliations:** ^1^ Department of Genomic Medicine, College of Medicine, Kaohsiung Medical University, Kaohsiung, Taiwan; ^2^ Department of Nursing, Shu-Zen College of Medicine and Management, Kaohsiung, Taiwan; ^3^ Department of Surgery, Division of General Surgery Medicine, Kaohsiung Medical University Hospital, Kaohsiung, Taiwan; ^4^ Cancer Center, Kaohsiung Medical University Hospital, Kaohsiung Medical University, Kaohsiung, Taiwan; ^5^ Graduate Institute of Medicine, College of Medicine, Kaohsiung Medical University, Kaohsiung, Taiwan; ^6^ Department of Surgery, Division of Gastroenterology and General Surgery, Kaohsiung Medical University Hospital, Kaohsiung Medical University, Kaohsiung, Taiwan; ^7^ Department of Internal Medicine, Division of Gastroenterology, Kaohsiung Medical University Hospital, Kaohsiung Medical University, Kaohsiung, Taiwan; ^8^ Department of Internal Medicine, Faculty of Medicine, College of Medicine, Kaohsiung Medical University, Kaohsiung, Taiwan; ^9^ Department of Nursing, Kaohsiung Medical University Hospital, Kaohsiung Medical University, Kaohsiung, Taiwan; ^10^ Department of Medical Research, Kaohsiung Medical University Hospital, Kaohsiung, Taiwan; ^11^ Graduate Institute of Clinical Medicine, College of Medicine, Kaohsiung Medical University, Kaohsiung, Taiwan; ^12^ Department of Surgery, Faculty of Medicine, College of Medicine, Kaohsiung Medical University, Kaohsiung, Taiwan; ^13^ Center for Biomarkers and Biotech Drugs, Kaohsiung Medical University, Kaohsiung, Taiwan

**Keywords:** hyperglycemia, colorectal cancer, miR-16, DM, prognosis

## Abstract

The high prevalence of type 2 diabetes mellitus in colorectal cancer patients is a crucial public health issue worldwide. The deregulation of microRNAs has been shown to be associated with the progression of CRC; however, the effects of high blood sugar levels on miR deregulation and, in turn, CRC remain unexplored. In this study, 520 CRC patients were classified into two groups according to their blood sugar levels (≧110 or <110 mg/dL). Clinicopathologic features, clinical outcomes, and serum miR-16 levels of the two groups were then analyzed, while cell cycles, cell proliferation, migration, and cellular miR-16 expression were investigated via D-(+)-glucose administration. Additionally, the target genes of miR-16 were identified. Through multivariate analysis, both the disease-free survival and overall survival of the CRC patients were found to be associated with the UICC stage, perineural invasion, and blood glucose levels (*P* < 0.05). Serum miR-16 levels were significantly lower in the high blood glucose patients than in the normal blood glucose patients (*P* = 0.0329). With D-(+)-glucose administration, the proliferation and migration of CRC cells *in vitro* increased remarkably (*P* < 0.05), while their accumulation in the G1 phase decreased significantly. Cellular miR-16 expression was suppressed by D-(+)-glucose administration. The expression levels of two target genes, *Myb* and *VEGFR2*, were affected significantly by miR-16, while glucose administration inhibited miR-16 expression and enhanced tumor cell proliferation and migration. Hyperglycemia can impact the clinical outcomes of CRC patients, likely by inhibiting miR-16 expression and the expression of its downstream genes *Myb* and *VEGFR2*.

## INTRODUCTION

Colorectal cancer (CRC) is a significant public health problem. Nearly one million new cases of CRC are diagnosed annually worldwide, and approximately half a million of these cases result in death [1]. Although radical surgical resection can be highly effective for localized diseases, 25-40% of patients develop recurrence/metastasis after surgery [2]. The recurrence of CRC is a time-limited phenomenon, and it has been shown that the length of patients' recurrence periods correlates strongly with the length of their survival periods [3, 4]. Presently, no ideal biomarker or indicator for predicting the recurrence/metastasis of CRC after operation exists [5, 6]. Continuous efforts have been made to enhance the methods of early tumor detection so as to assist physicians in intensifying surveillance and therapeutic strategies, thereby improving the patients' prognoses [5, 7, 8].

The proper control of metabolic homeostasis is crucial to maintaining human physiology and health. Relatedly, systematic review reports have demonstrated that fatty-acid metabolism plays a significant role in the tumorigenesis of human CRC [9]. Moreover, increased glycolytic activity among malignant tumor cells has been demonstrated both *in vivo* and *in vitro* [10], while diabetic people have been shown to have an increased risk of CRC as compared to non-diabetics [11]. Mature microRNAs (miRs) that function as translational repressors have recently been found to be key regulators of metabolism and tumorgenesis [12–17]. Tumor-derived microvesicles are enriched with bioactive molecules, and plasma miRs. Microvesicles miRs are protected from endogenous RNase activity and are involved in cancer progression and immune-response inhibition [18]. Circulating miRs have been shown to be promising circulating biomarkers for CRC detection and progression [19–22]. Although several studies have focused on the deregulation of miRs, involving either the pathogenesis of metabolic disorders [12, 13] or CRC carcinogenesis [14–17], studies in the deregulation of miRs involving glucose metabolism and CRC recurrence/prognosis are sparse. Previous studies have shown the downregulation of the serum miR-16 family in patients with metabolic syndrome [23, 24] and the downregulation of miR-195 in patients with poor prognoses in CRC [25]. Consequently, this study explores the correlation between the glycolysis-related miRs/relevant miRs target genes and CRC relapse/prognosis.

In the current study, we attempted to determine the correlations, if any, between the serum blood sugar levels and clinical outcomes of CRC patients. Furthermore, we investigated *in vitro* whether high serum blood sugar levels could affect the prognoses of CRC patients through miRs deregulation and the modulation of miRs downstream genes.

## RESULTS

### Demographic data and clinical outcomes

The clinicopathologic features of 520 independent CRC patients (312 in the normal glucose group vs. 208 in the high glucose group) are summarized in Table 1. The median blood sugar level of the patients was 105 mg/dL, with a range from 70 to 395. The median age of the patients was 66 years, with a range from 24 to 89. Patients in the DM history group had significantly higher blood sugar levels than those in the non-diabetes group (*P* < 0.0001, Table 1). In addition, the results in Table 1 also indicate significant differences in tumor size (*P* = 0.042), age (*P* = 0.005), and the presence of perineural invasion (*P* = 0.022) between the normal blood sugar group (< 110 mg/dL) and the high blood sugar group (≧110 mg/dL), but no significant differences in terms of other clinicopathologic features, including gender (*P* = 0.942), tumor location (*P* = 0.874), tumor invasion depth (*P* = 0.282), lymph node metastasis (*P* = 0.288), stage (*P* = 0.413), vascular invasion (*P* = 0.102) and differentiation grade (*P* = 0.964).

**Table 1:** Baseline characteristics of 520 colorectal cancer patients based on serum blood sugar concentrations using univariate analysis

Variables		Serum blood sugar^1^ < 110 mg/dL (*n* = 312) No(%)	Serum blood sugar^1^ ≧110 mg/dL (*n* = 208) No(%)	*P* value
**Age** (y) (Mean ± SD		63.32 ± 12.49	66.42 ± 12.30	0.005
**DM^2^**	no	278 (89.10)	107 (51.44)	<0.0001
	yes	34 (10.90)	101 (48.56)	
**Gender**	female	130 (41.67)	86 (41.35)	0.942
	male	182 (58.33)	122 (58.65)	
**Tumor size**	< 5 cm	196 (62.82)	112 (53.85)	0.042
	≧5 cm	116 (37.18)	96 (46.15)	
**Location**	colon	223 (71.47)	150(72.12)	0.874
	rectum	89 (28.53)	58 (27.88)	
**Invasion depth**	T1	19 (6.09)	13 (6.25)	0.282
	T2	51 (16.35)	24 (11.54)	
	T3	227 (72.76)	155 (74.52)	
	T4	15 (4.81)	16 (7.69)	
**Lymph node metastasis**	No	188 (60.26)	115 (55.56)	0.288
	Yes	124 (39.74)	92 (44.44)	
**Stage**	I	53 (16.99)	27 (12.98)	0.413
	II	133 (42.63)	89 (42.79)	
	III	126 (40.38)	92 (44.23)	
**Vascular invasion**	No	246 (78.85)	151 (72.60)	0.102
	Yes	66 (21.15)	57 (27.40)	
**Perineural invasion**	No	229 (73.40)	133 (63.94)	0.022
	Yes	83 (26.60)	75 (36.06)	
**Grade**^3^	WD	12 (3.85)	9 (4.33)	0.964
	MD	267 (85.58)	177 (85.10)	
	PD	33 (10.58)	22 (10.58)	
**Type**^4^	A	301 (96.47)	195 (93.75)	0.352
	M	9 (2.88)	11 (5.29)	
	S	2 (0.64)	2 (0.96)	

1 AC (Ante cibum= before eating) serum blood sugar before surgery

2 Confirmed diagnosis of diabetes mellitus (DM) before surgery

3 WD: Well differentiated; MD: Moderately differentiated; PD: Poorly differentiated

4 A: Adenocarcinoma; M: Mucinous carcinoma; S: Signet-ring cell carcinoma.

Further stratification of CRC patients according to DM history status (Table 2) showed that patients in the normal blood glucose level group with or without a DM history had a lower percentage of relapse compared to patients in the high blood glucose level group (*P* = 0.0001 and 0.0115, respectively). Patients without a history of DM and who maintained a blood glucose level below 110 mg/dL had a better overall survival rate than those with a high blood glucose level (*P* = 0.0004, Table 2), but no significant differences between high and normal blood glucose groups among patients with DM were observed (*P* = 0.5225, Table 2).

**Table 2:** Correlation between postoperative relapse, survival and diabetes mellitus (DM) history in 520 UICC^1^ stage I-III colorectal cancer patients

Variables		No DM history (N = 385)		*P* value	DM history (N = 135)		*P* value
		Blood sugar < 110 mg/dL (N = 278) No(%)	Blood sugar ≧ 110 mg/dL (N = 107) No(%)		Blood sugar < 110 mg/dL (N = 34) No(%)	Blood sugar ≧ 110 mg/dL (N = 101) No(%)	
**Relapse^2^**	**No**	192 (69.06)	51 (47.66)	0.0001	28 (82.35)	60 (59.41)	0.0115
	**Yes**	86 (30.94)	56 (52.34)		6 (17.65)	41 (40.59)	
**Overall Survival**	**Yes**	230 (82.73)	70 (65.42)	0.0004	28 (82.35)	78 (77.23)	0.5225
	**No**	48 (17.27)	37 (34.58)		6 (17.65)	23 (22.77)	

1 UICC: Union for International Cancer Control

2 Local recurrence or distant metastases after surgery

### Impact on disease-free survival (DFS) and overall survival (OS)

Using Cox regression hazard analysis, the prognostic factors for DFS and OS for CRC patients was shown (Table 3). Multivariate analyses showed the advanced UICC stage (*P* < 0.0001, HR: 2.200, 95% CI: 1.618-3.005. Table 3), the presence of perineural invasion (*P* = 0.0003, HR: 1.773, 95% CI: 1.299-2.414, Table 3), DM history (*P* = 0.025, HR: 0.660, 95% CI: 0.453-0.950, Table 3), and high blood sugar levels (*P* < 0.0001, HR: 2.206, 95% CI: 1.467-2.788, Table 3) to be significant independent poor prognostic factors for DFS. For OS, the advanced UICC stage (*P* < 0.0001, HR: 2.294, 95% CI: 1.536-3.462, Table 3), tumor size ≧5 cm (*P* = 0.050, HR: 1.467, 95% CI: 1.000-2.156, Table 3), presence of perineural invasion (*P* = 0.016, HR: 1.656, 95% CI: 1.101-2.480, Table 3), and high blood sugar levels (*P* = 0.002, HR: 1.917, 95% CI: 1.265-2.896, Table 3) to be significant independent poor prognostic factors. DM status was a significant independent prognostic factor for DSF, however, high blood glucose level was a significant independent prognostic factor for both DSF and OS. Blood sugar level was considered to be more significant than DM status for the clinical outcome of CRC patients.

**Table 3:** Correlation between disease-free survival (DFS) and overall survival (OS) with clinicopathologic features of 520 UICC^1^ stage I-III CRC patients using a Cox regression analysis

Variables	Number	DFS				OS			
		Univariate analysis		Multivariate analysis		Univariate analysis		Multivariate analysis	
		*P* value	HR^2^ (95% CI^3^)	*P* value	HR^2^ (95% CI^3^)	*P* value	HR^2^ (95% CI^3^)	*P* value	HR^2^ (95% CI^3^)
**Sex (Male/Female)**	304/216	0.651	1.069 (0.801-1.434)	0.884	1.022 (0.762-1.379)	0.258	1.242 (0.854-1.827)	0.391	1.184 (0.807-1.756)
**Age (≥65/< 65) years**	281/239	0.149	0.810 (0.608-1.078)	0.497	0.902 (0.670-1.214)	0.687	0.927 (0.642-1.341)	0.705	1.077 (0.734-1.583)
**Invasive depth (T4 + T3/T2 + T1)**	413/107	< 0.0001	2.348 (1.539-3.766)	0.103	1.461 (0.929-2.403)	0.005	2.094 (1.239-3.830)	0.602	1.170 (0.663-2.209)
**Stage (III/II+ I)**	218/302	< 0.0001	2.569 (1.923-3.448)	< 0.0001	2.200 (1.618-3.005)	< 0.0001	2.719 (1.869-4.003)	< 0.0001	2.294 (1.536-3.462)
**Tumor size (≥5/< 5)cm**	212/308	0.061	1.317 (0.987-1.753)	0.258	1.188 (0.881-1.599)	0.012	1.603 (1.110-2.318)	0.050	1.467 (1.000-2.156)
**Vascular invasion (yes/no)**	123/397	< 0.0001	1.877 (1.378-2.531)	0.492	1.123 (0.805-1.555)	< 0.0001	2.370 (1.617-3.438)	0.076	1.460 (0.961-2.203)
**Perineural invasion (yes/no)**	158/362	< 0.0001	2.218 (1.661-2.953)	0.0003	1.773 (1.299-2.414)	0.0001	2.074 (1.430-2.996)	0.016	1.656 (1.101-2.480)
**Blood sugar^4^ (≧110/< 110) mg/dL**	208/312	< 0.0001	1.782 (1.339-2.373)	< 0.0001	2.026 (1.467-2.788)	0.002	1.785 (1.236-2.585)	0.002	1.917 (1.265-2.896)
**DM history(yes/no)**	135/385	0.843	0.967 (0.689-1.334)	0.025	0.660 (0.453-0.950)	0.972	0.993 (0.641-1.493)	0.103	0.679 (0.418-1.079)

* The multiple logistic regressions with/without adjustment of age, sex, stage, tumor size, location, invasive depth, vascular invasion, perineural invasion and serum blood sugar of CRC patients.

1 UICC: Union for International Cancer Control

2 HR:Harzards ratio

3 95% CI: 95% Confidence interval

4 AC (Ante cibum= means before eating) serum blood sugar before surgery

### Effects of differing glucose concentrations on colon cancer cell proliferation

Three thresholds of D-(+)-glucose concentrations (i.e., a baseline concentration of 5 mM (90 mg/dL) and the two higher concentrations of 10 mM (181 mg/dL) and 15 mM (271 mg/dL)) were used to examine the role of glucose administration on the tumorigenesis of colon cancer cells. Caco2, SW480, and SW620 cells were treated for 24 h under the varying D-(+)-glucose concentrations (5, 10 and 15 mM). As shown in Figure 1A, the proliferation rate of Caco2 and SW620 by 10 mM of D-(+)-glucose administration increased significantly at 24 h (*P* = 0.0053 and 0.0035, respectively), compared to the rate by 5 mM of D-(+)-glucose administration. The proliferation rate of Caco2 and SW620 by 15 mM of D-(+)-glucose administration increased significantly at 24 h compared to the rate at 5 mM of D-(+)-glucose administration (both *P* < 0.0001). The proliferation rate of SW480 cells under various D-(+)-glucose concentrations did not vary significantly at 24 h (Figure 1A). Using flow cytometry, we further examined the influence of the various D-(+)-glucose concentrations on cell cycles. The rate of growth of SW480 cells was slower, and their accumulation in the G1 phase in 5 mM of D-(+)-glucose was higher than the accumulation rates for the other two cell lines (83.57% in SW480 (Supplementary Figure S1) *vs.* 55.15% in Caco2 or 51.15% in SW620 (Figure 1B)).

**Figure F1:**
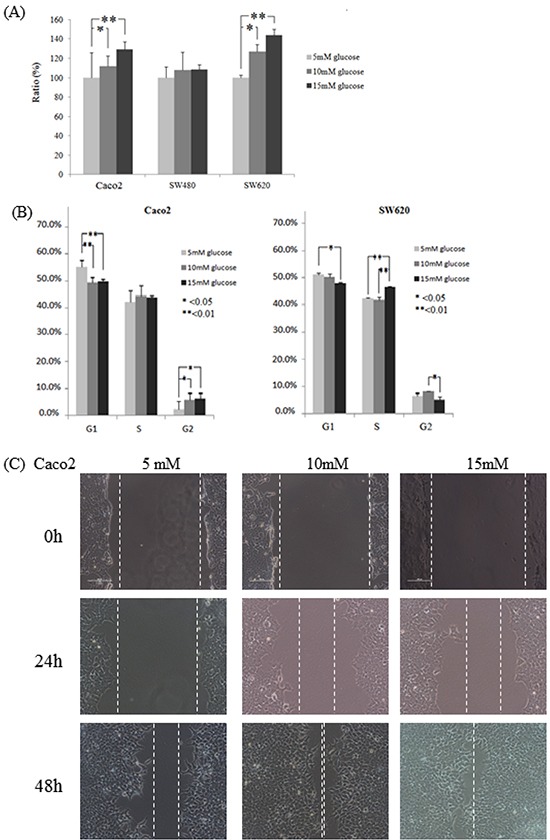
Colon cancer cell lines Caco2, SW480, and SW620 incubated in varying glucose conditions affects cell cycle, proliferation, and migration **A.** Through the WST-1 assay, cell proliferation of Caco2 and SW620 increased significantly for 24 h incubation in a high glucose concentration. Compared to that of 5 mM of D-(+)-glucose administration, the proliferation rate of Caco2 in 10 mM and 15 mM of D-(+)-glucose administration increased significantly (*P* = 0.0053 and *P* < 0.0001, respectively). The proliferation rate of SW620 by 10 mM and 15 mM of D-(+)-glucose administration increased significantly at 24 h (*P* = 0.0035 and *P* < 0.0001, respectively). **B.** For Caco2 and SW620 cells, the accumulation in the G1 phase decreased significantly after 24 h of incubation in varying glucose conditions, while the accumulation in the G2 phase increased significantly only under the high glucose conditions. Compared to the 5 mM D-(+)-glucose concentration, the accumulation of Caco2 cells in the G1 phase in the 10 mM and 15 mM D-(+)-glucose concentrations was decreased significantly at 24 h (*P* = 0.0002 and 0.0014, respectively), while the accumulation in the G2 phase was increased significantly in those higher concentrations (*P* = 0.0186 and 0.0152, respectively). For SW620 cells, the accumulation in the G1 phase was 51.15% in the 5 mM D-(+)-glucose concentration and 50.02% in the 10 mM D-(+)-glucose concentration (*P* = 0.0315 and 0.089, respectively) *vs*. 47.93% in the 15 mM of D-(+)-glucose concentration, while the accumulation in the G2 phase was 6.53% in the 5 mM concentration (*P* = 0.223 and 0.032, respectively) *vs*. 8.20% in the 10 mM concentration and 5.54% in the 15 mM concentration. **C.** The cell migration ability of Caco2 cells was increased in the high glucose concentrations as indicated by narrower gaps at 48 h. After 24 h of incubation, the narrowest gap distances decreased at the higher glucose concentrations (but not reach significantly different, both *P* > 0.05). After 48 h, the gap distances narrowed down significantly at the higher concentrations (0.48 mm in 5 mM of D-(+)-glucose *vs*. 0.08 mm in 10 mM of D-(+)-glucose and 0 mm in 15 mM of D-(+)-glucose; *P* = 0.017 and 0.002, respectively).

### Effects of differing glucose concentrations on the colon cancer cell cycle

For Caco2 cells after 24 h of incubation, accumulation in the G1 phase decreased significantly at the higher glucose concentrations (55.15% in 5 mM of D-(+)-glucose *vs*. 49.22% in 10 mM of D-(+)-glucose and 49.78% in 15 mM of D-(+)-glucose; *P* = 0.0002 and 0.0014, respectively (Figure 1B)) while accumulation in the G2 phase was increased significantly at the higher concentrations (2.52% in 5 mM of D-(+)-glucose *vs*. 6.22% in 10 mM of D-(+)-glucose and 6.54% in 15 mM of D-(+)-glucose; *P* = 0.0186 and 0.0152, respectively (Figure 1B)). For SW620 cells after 24 h of incubation, a significantly decreased accumulation in the G1 phase at the higher concentrations (51.15% in 5 mM of D-(+)-glucose and 50.02% in 10 mM of D-(+)-glucose *vs*. 47.93% in 15 mM of D-(+)-glucose, *P* = 0.0315 and 0.089, respectively; Figure 1B) was found. A significantly increased accumulation in the S phase at the highest concentration (42.32% in 5 mM of D-(+)-glucose and 41.79% in 10 mM of D-(+)-glucose *vs*. 46.68% in 15 mM of D-(+)-glucose, *P* = 0.0129 and 0.0094, respectively (Figure 1B)) and a significantly decreased accumulation in the G2 phase at the highest concentration (6.53% in 5 mM of D-(+)-glucose *vs*. 8.20% in 10 mM of D-(+)-glucose and 5.54% in 15 mM of D-(+)-glucose, *P* = 0.223 and 0.032, respectively (Figure 1B)) were found. The two colon cancer cell lines (Caco2 and SW620) were shown to have significantly decreased accumulations in the G1 phase when subjected to the high D-(+)-glucose concentrations.

Due to the slow growth rate of SW480 cells, the incubation time for the SW480 cell cycle experiment was extended to 48 h. After 48 h of incubation (Supplementary Figure S1A), accumulation in the G1 phase decreased significantly in the higher concentrations (83.57% in 5 mM of D-(+)-glucose *vs*. 79.63% in 10 mM of D-(+)-glucose and 79.35% in 15 mM of D-(+)-glucose, *P* = 0.0079 and 0.0054, respectively), while the accumulation in the G2 phase increased significantly (1.52% in 5 mM of D-(+)-glucose *vs*. 4.19% in 10 mM of D-(+)-glucose and 4.36% in 15 mM of D-(+)-glucose, both *P* < 0.0001).

### Effects of differing glucose concentrations on cell migration ability

For the Caco2 cells, wound healing analysis indicated that the gap distances for 5mM, 10mM, and 15mM glucose concentrations at 0 h were 1.84 mm, 1.84 mm, and 1.88 mm, respectively. After 24 h of incubation, the narrowest gap distances decreased to 1.64 mm in 5 mM of D-(+)-glucose, 0.76 mm in 10 mM of D-(+)-glucose, and 0.72 mm in 15 mM of D-(+)-glucose (both *P* > 0.05). After 48 h, the gap distances narrowed down to 0.48 mm in 5 mM of D-(+)-glucose *vs*. 0.08 mm in 10 mM of D-(+)-glucose and 0 mm in 15 mM of D-(+)-glucose (*P* = 0.017 and 0.002, respectively) (Figure 1C). For the SW480 and SW620 cells, cell migration was also examined in terms of the varying concentrations of D-(+)-glucose. The SW480 cells were shown to react in one particular way by floating into the media rather than attaching to a new area of the plate (Supplementary Figure S2A), while the SW620 cells were shown to react in another way by piling on top of each other and forming a large lump (Supplementary Figure S2B). These results show that while the colon cancer cells clearly exhibited increased migration ability when subjected to high concentrations of glucose, the SW480 and SW620 cell lines were not suitable for use in wound healing analysis.

### Decreased circulating miR-16 expression in high blood sugar group

**Figure 2:**
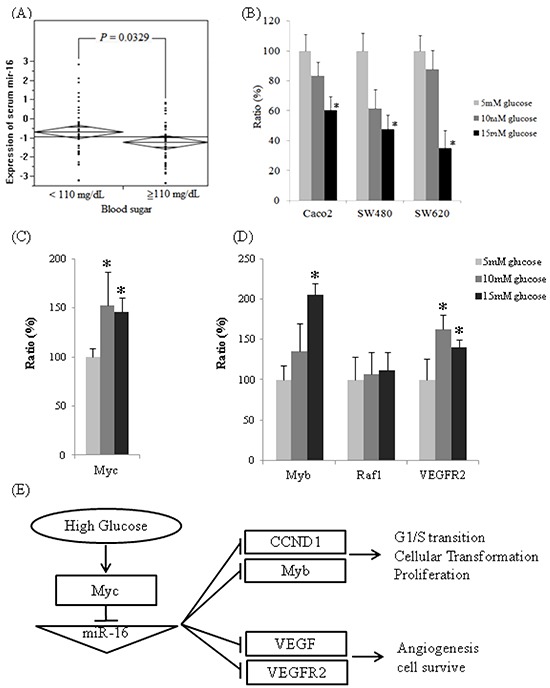
The expressions of miR-16 are downregulated in high glucose conditions *in vivo* and *in vitro* and the expression of miR-16 related mRNAs **A.** Reverse transcription-qPCR analysis of serum miR-16 expression level by normalizing to *C. elegans* synthetic *lin-4* miR expression. **B.** Quantitative analysis of miR-16 expression in three colon cancer cell lines after incubation in three separate D-(+)-glucose concentrations (5, 10, and 15 mM) for 24 h. The relative expression levels of miR-16 were normalized to U6b expression. **C.** The mRNA expression levels of *Myc*, as determined by qPCR, were significantly higher in 10 mM of D-(+)-glucose (152%, *P* < 0.0001, dark gray) and 15 mM of D-(+)-glucose (145%, *P* = 0.0005, black) compared to the one in 5 mM of D-(+)-glucose (100%, light gray) in Caco2. **D.** The mRNA expression levels of *Myb*, *Raf-1*, and *VEGFR2*, as determined by qPCR. Compared to the one in 5mM of D-(+)-glucose, mRNA levels of *Myb* were higher in 10mM of D-(+)-glucose (135%, *P* = 0.060) and 15 mM of D-(+)-glucose (206%, *P* < 0.0001). The expression levels of *Raf-1* mRNA were not significantly different in 10 mM of D-(+)-glucose (107%, *P* = 0.621) and 15 mM of D-(+)-glucose (112%, *P* = 0.357). Compared to 5 mM of D-(+)-glucose, the mRNA levels of *VEGFR2* were significantly higher in 10 mM of D-(+)-glucose (163%, *P* = 0.0002) and 15 mM of D-(+)-glucose (140%, *P* = 0.0015). **E.** The proposed mechanism affecting colon cancers by glucose administration through miR-16 regulation.

Preoperative serum miR-16 levels varied significantly between the low glucose (< 110mg/dL, *N*=46) and high glucose (≧110mg/dL, *N*=44) patients. The mean of log_10_ (2^−ΔCt^) was −0.68 in the normal glucose group and −1.22 in the high glucose group (Figure 2A). Therefore, serum miR-16 levels decreased 3.5-fold in high glucose patients compared to those in the normal glucose group (*P* = 0.0329).

### Cellular miR-16 expression levels under various glucose concentrations

The expression of cellular miR-16 was quantified by qPCR in the CRC samples (Caco2, SW480, and SW620) that were cultured in different glucose concentrations for 24 h incubation. The expressions of miR-16 decreased in three colon cancer cell lines in high-glucose conditions compared with the 5 mM of D-(+)-glucose condition (Figure 2B); the expressions of miR-16 for Caco2 cells decreased to 83.42% in 10 mM of D-(+)-glucose (*P* = 0.420) and to 60.27% in 15 mM of D-(+)-glucose (*P* = 0.027); the expressions of miR-16 for SW480 cells decreased to 61.42% in 10 mM of D-(+)-glucose (*P* = 0.066) and to 47.75% in 15 mM of D-(+)-glucose (*P* = 0.007); the expressions of miR-16 for SW620 cells decreased to 87.74% in 10 mM of D-(+)-glucose (*P* = 0.565) and to 34.97% in 15 mM of D-(+)-glucose (*P* < 0.0001). These results imply that the invasive colon cancer cell line was affected more significantly by high blood sugar than was the less invasive one.

### The results of target genes prediction and mRNA quantitative assay

Our experiments showed that high glucose conditions can enhance cell proliferation in 3 colon cancer cell lines and decrease the population of arrested cells in the G0/G1 phase. We identified miR-16 target genes that may elucidate the proliferation and anti-oncogenic effects by bioinformatic analysis. Multifunctional, transcription factors, *Myb* and *Raf1*, which play a role in cell cycle progression and cellular transformation, and angiogenesis-related and cell-survival genes, *VEGF* and *VEGFR2*, were predicted.

After 24 h incubation in high glucose conditions, the mRNA levels of *Myc* for Caco2 cells increased significantly to 152% in 10 mM of D-(+)-glucose (*P* = 0.0001) and 145% in 15 mM of D-(+)-glucose (*P* = 0.0005), compared with the baseline condition (5 mM of D-(+)-glucose, Figure 2C). When cellular *Myc* mRNA were overexpressed under high glucose circumstances, the cellular miR-16 expression is suppressed, and subsequently miR-16 target genes, *Myb* and *VEGFR2* mRNA were overexpressed (Figure 2D). Compared with 5 mM of D-(+)-glucose, the *Myb* mRNA levels increased to 135% in 10 mM glucose (*P* = 0.060) and 206% in 15mM glucose (*P* < 0.0001), and the *VEGFR2* mRNA levels increased significantly to 163% in 10 mM of D-(+)-glucose (*P* = 0.0002) and 140% in 15 mM of D-(+)-glucose (*P* = 0.0015). The high glucose concentrations slightly increased *Raf-1* mRNA expression to 107% in 10 mM of D-(+)-glucose (*P* = 0.621) and 112% in 15 mM of D-(+)-glucose (*P* = 0.357).

### Survival analysis

**Figure 3:**
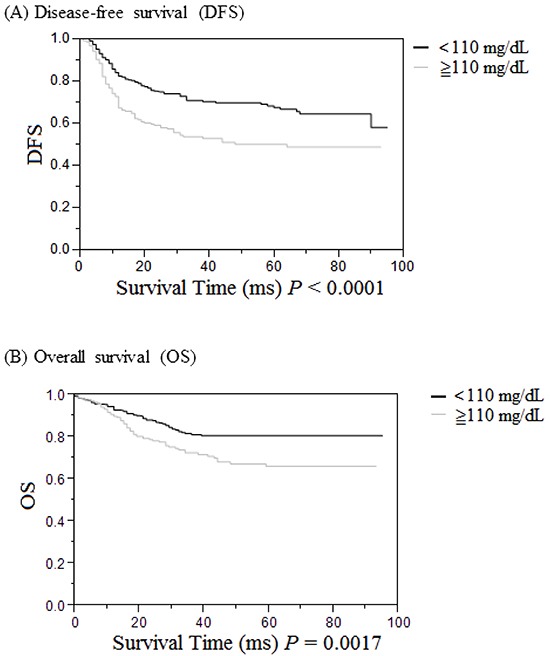
Cumulative survival rates of the 520 UICC stages I to III patients with colorectal cancer (CRC) as assessed by the Kaplan-Meier method, and differences in survival rates analyzed by the log-rank test **A.** Disease-free survival in CRC patients was significantly lower in the high serum glucose group (≧110 mg/dL, gray line) than in the low serum glucose group (<110 mg/dL, black line) (*P* < 0.0001). **B.** Overall survival in CRC patients was significantly lower in the high serum glucose group (≧110 mg/dL, gray line) than in the low serum glucose group (< 110 mg/dL, black line) (*P* = 0.0017).

DFS and OS of the 520 UICC stage I to III CRC patients were assessed by the Kaplan-Meier method (Figure 3). Both DFS (*P* < 0.0001, Figure 3A) and OS (*P* = 0.0017, Figure 3B) were significantly poorer in the high blood glucose group (≧110 mg/dL) compared to those in the normal blood glucose group (< 110 mg/dL).

## DISCUSSION

CRC is a disease with a complex etiology, and the mechanisms underlying this pathophysiology are not yet fully understood. Investigations of epigenetic modifications have accounted for the majority of research into complex diseases, and studies of this type have indicated that miRs could possibly serve as potential biomarkers of cancer [5, 7, 32]. The most fundamental metabolism alteration in CRC cells is the increase in glycolysis [10, 33]. Glucose may drive cancer not only by modifying miR expression levels but also by activating the mammalian target of rapamycin, which can increase protein synthesis and cellular growth [34–38]. In this study, we observed a higher incidence of postoperative recurrence in hyperglycemia patients and confirmed the vital role of miR-16 in hyperglycemic CRC patients. Through a series of *in vitro* studies, we showed that high glucose conditions can enhance cell proliferation in 3 colon cancer cell lines, decrease the population of arrested cells in the G0/G1 phase, as well as increase cell migration. Following glucose administration, miR-16 expression was inhibited through the overexpressed *Myc* gene, as in previous reports [39, 40]. Moreover, the expressions of *Myb* and *VEGFR2* mRNA, which are target genes of miR-16, were shown to be upregulated.

This study offers biological plausibility for the hypothesis that hyperglycemia decreases serum miR-16 expression in CRC patients. Specifically, the experimental results showed that subjecting the Caco2, SW480, and SW620 colon cancer cells to high concentrations of glucose can increase the proliferation of the cells and decrease their accumulation in the G1 phase. miR-15a and miR-16-1 as the miR-15a/16-1 cluster are located on chromosome13q14 and the region was deleted in more than half of chronic lymphocytic leukemia cases and accelerated the proliferation of human B cells by modulating the expression of genes controlling cell-cycle progression [41]. Previous studies have shown that miR-16 partially induces cell-cycle arrest by regulating multiple cell-cycle genes, including *cyclin D1*, *cyclin D3*, *cyclin E1*, and *CDK6*, and by preventing cells from entering the S phase, causing an accumulation of cells in the G0/G1 phase [42]. The miR-16 family inhibits cellular growth and cell cycle progression, and triggers the G0/G1 accumulation phenotype in diverse cell lines, including HCT116, DLD-1, A549, MCF7, and Tov21G cells [43, 44].

Through a literature review, we have learned that multiple targets regulated by an individual miR can act in a coordinated manner to regulate the same biological process [43]. The vascular endothelial growth factor (VEGF) plays an important role in tumor development, growth, and metastasis [45]. Dejean *et al.* have shown that downregulation of miR-16 induces VEGF expression, tumor angiogenesis, and growth in anaplastic lymphoma kinase (ALK)-positive anaplastic large-cell lymphomas [46]. Vascular endothelial growth factor receptor (VEGFR) can be expressed on the cell surface of several tumor types [47], and we have shown that a high glucose level can enhance *VEGFR2* mRNA expression by suppressing miR-16 expression. Overexpressed VEGF not only stimulates angiogenesis for tumor growth but also acts with VEGFR on the tumor-cell surface as an autocrine growth factor to enhance the tumor [47]. We propose that downregulated miR-16 results in upregulating two VEGF pathway proteins, VEGF and VRGFR2, simultaneously and enhances tumor growth by both VEGF autocrine and angiogenesis. c-Myb portion, a transcription factor, is highly expressed in the gastrointestinal tract [48]. Overexpression of c-Myb increases the tumorigenesis of colon-cancer cells and poor prognosis for CRC patients [49, 50]. In high glucose conditions, miR-16 expression were suppressing and resulted in overexpressing of miR-16 target genes *c-Myb* and *VEGFR2* and led to colon cancer cell proliferation and migration.

The meta-analysis showed a positive correlation of diabetes mellitus and increased risk of CRC [11]. The present data shows that stage I-III CRC patients with high blood sugar levels have a lager tumor size, more perineural invasion, and markedly poor prognosis, including a poor DFS and OS. We further stratified the CRC patients according to their DM status and found that those patients either with or without DM history but whose blood sugar levels were below 110mg/dL had a lower incidence of relapse. Previous studies indicate that miRs in the plasma microvesicles might regulate the progression of cell-cycle proteins [51, 52]. Since CRC patients with hyperglycemia are observed to express lower serum miR-16 levels, and they are more prone to having poor clinical outcomes, miR-16 has an antitumorigenesis effect on colon cancer cells. This study suggests that circulating miR-16 can serve as a novel and convenient biomarker for post-operative surveillance, so regular serum miR-16 expression tests are recommended. However, further large-scale follow-up studies are needed to demonstrate this hypothesis.

In this study, we only included the fasting blood sugar levels, which reflect daily blood glucose level, but which also might be prone to bias. HbA1C levels can be tested at any time, notwithstanding the duration of fasting or the type of prior meal, and HbA1c levels reflect blood glucose levels over the past 6 to 8 weeks [53]. The HbA1c levels may thus be more crucial in terms of their prognostic impact for CRC patients, but we did not have HbA1c data for every patient in the current study. At present, however, we are conducting another study that combines fasting plasma glucose and HbA1C data to determine if HbA1C is more crucial in terms of its prognostic impact for CRC patients.

In summary, data from this study show that miR-16 has anti-oncogenic effects, including the inhibition of colon-cancer cell proliferation and migration. The association between hyperglycemia, decreased levels of miR-16, and prognosis in CRC patients suggest that blood sugar levels and serum miR-16 of patients are potential surrogate biomarkers for identifying high-risk stage I-III CRC patients after radical resection.

## MATERIALS AND METHODS

### Patients and tumor samples

In this retrospective study, CRC patients for whom fasting blood glucose levels were not determined, those with incomplete medical records, and those with stage IV disease or without the informed consent were excluded. Demographic data were obtained for 520 patients with primary CRC at the American Joint Commission on Cancer/International Union Against Cancer (AJCC/UICC) [1] stages I to III between January 2005 and July 2011; each of the patients enrolled in the study signed an informed consent indicating their agreement to participate. Impaired carbohydrate metabolism was defined as one of the ADA 2003 diagnostic criteria (diabetes: fasting plasma glucose [FPG]: ≥126 mg/dL; impaired fasting glucose [IFG]: 110-125 mg/dL; normal: < 110 mg/dL) [26, 27]. The blood sugar levels of all 520 CRC patients were determined using blood samples collected when the patients had achieved fasting status, and of these 520 patients, 312 had fasting glucose levels between 70 and 109 mg/dL (less than 110 mg/dL: normal glucose group) and 208 patients had levels between 110 and 395 mg/dL (no less than 110 mg/dL: high glucose group). Furthermore, serum miR-16 levels were measured in 90 patients (46 samples from the normal glucose group and 44 samples from high glucose group). The diagnoses of diabetes mellitus (DM) were made based on the DM chart history or by taking anti-hyperglycemic agents for DM. All subjects were unrelated ethnic-Chinese residents in Taiwan. Each patient provided written informed consent for collecting their clinical samples and to publish their case details, and all patient data were anonymized. The study protocol was approved by the Kaohsiung Medical University Hospital Institutional Review Board (Protocol Number: KMUHIRB-2012-04-02(I)). All patients received follow ups until either their death or December 2012. The median follow-up time was 37 months (range: 3 to 95 months). Disease-free survival (DFS) was defined as the time between primary surgery and the recurrence of colon cancer or the last follow-up appointment. Overall survival (OS) was defined as the elapsed time between primary surgery and death from any cause or the last follow-up appointment.

### Cell culture

The Caco2 and SW480 colon cancer cell lines were established from primary adenocarcinoma of the colon, and the SW620 cell line was established from a lymph node metastasis of the same patient. The human colon carcinoma cell lines Caco2, SW480, and SW620 (ATCC, Manassas, VA, USA) were cultured in low glucose DMEM (5 mM D-(+)-glucose, Gibco-BRL, Gaithersburg, MD, USA) supplemented with 10% fetal calf serum (FCS; Gibco-BRL) and 100 U/mL of penicillin, as described previously [28]. The cells were maintained at 37°C in an atmosphere of 5% CO_2_.

### Analysis of cell proliferation

CRC cell lines Caco2, SW480, and SW620 were seeded in 96-well plates in the baseline glucose DMEM (D-(+)-glucose 5 mM, Gibco-BRL) as described above. Cells were treated (4 wells per treatment) in various D-(+)-glucose concentrations (5, 10, or 15 mM) for 22 h. To determine cell proliferation, cells were further incubated with 1/10 volume of WST-1 reagent (Roche Diagnostics Corp., Indianapolis, IN, USA) for 2 h at 37°C before absorbance was quantified using a spectrophotometer at 450 nm.

### Analysis of cell cycle

Colon cancer cell lines Caco2, SW480, and SW620 were seeded in 6-well plates as described above. Caco2 and SW620 cells were incubated (3 wells per treatment) for 24 h with various D-(+)-glucose concentrations (5, 10, or 15 mM) and cell cycles were quantified using propidium iodide (PI, Sigma-Aldrich Co, MO, USA) staining and subsequently analyzed using a FACScan cytofluorimeter (Becton Dickinson, NJ, USA) with CellQuest software (BD Biosciences), according to the manufacturer's instructions. Since SW480 cells were slower to grow than the other cell lines in the proliferation assay, SW480 cells were chosen and treated with different glucose concentrations (5, 10, or 15 mM) for 48 h.

### Wound healing assay

Colon cancer cell lines, Caco2, SW480, and SW620 were seeded in 6-well plates as described above until the cells formed a monolayer. Then, a wound was created by manual scraping with a 200-ml micropipette tip. The culture medium was then replaced with DMEM containing various D-(+)-glucose concentrations (5, 10, or 15 mM), and wound closure was monitored and photographed at various time points (0, 24, and 48 h) under a microscope.

### Serum preparation and RNA extraction

The venous blood was obtained prior to the operation. The blood samples were centrifuged at 3000 rpm for 15 min, and the serum was aliquoted into 1.7-mL eppendorf tubes. In the absence of well-documented, stably expressed endogenous circulating miRs as normalization controls, *C. elegans* synthetic *lin-4* miR (*Cel-lin*-4, Part Number: 4398988, Invitrogen, Carlsbad, CA, USA), which had been added to the serum preparation prior to RNA extraction, was used as a normalization control, following the procedure described in earlier studies [29, 30]. For the isolation of RNA from the serum, 300 μL of serum were homogenized in 900 μL of Trizol LS, according to the manufacturer's instructions (Invitrogen), with minor modifications: 6 μL of 1 nM *Cel-lin-4* were added to the serum samples. Then, 250 μL of chloroform were added to the sample, and the mixed solution was centrifuged. After an additional chloroform extraction and precipitation with isopropanol, the pellet was washed twice by centrifugation with 70% ethanol. The RNA pellet was dried for 10 min at room temperature and dissolved in 30 μL of distilled water. DNase treatment (Qiagen) was executed to remove any DNA contamination.

### Serum miR-16 expression levels of CRC patients

To measure circulating miR-16 expression levels, miR-16 cDNA was synthesized from 20 ng of total RNA with a unique primer (Applied Biosystems Inc., CA, USA). TaqMan miR RT-qPCR assay (Applied Biosystems Inc.) was used to quantify the levels of serum miR-16. The relative expression levels of miR-16 in the serum were normalized to that of *Cel-lin-4* using the equation log_10_ (2^−ΔCt^), where ΔCt=(Ct_miR-16_–Ct_Cel-lin-4_). The mean and standard deviation (SD) values of log_10_ (2^−ΔCt^) were calculated.

### Colon cancer cellular miR-16 expression levels

Approximately 10^7^ cells were lysed in 1 mL of TRIzol reagent (Invitrogen), according to the manufacturer's instructions. Total RNA, including mRNA and miR, was purified with Qiagen RNAeasy Columns (Qiagen, Hamburg, Germany). For measuring the miR-16 expression level of CRC cells, miR-16 cDNA was synthesized and TaqMan miR RT-qPCR assay was used, as previously described. The relative expression levels of miR-16 were normalized to that of U6b using the equation: log_10_ (2^−ΔCt^), where ΔCt=(Ct_miR-16_–Ct_U6b_). The mean and standard deviation (SD) values of log_10_ (2^−ΔCt^) were calculated.

### Target genes prediction

To investigate the biological functions of the miR-16, its target genes were searched by using several miR target prediction programs [31], which include TargetScanS (http://genes.mit.edu/targetscan/), miRDB (http://mirdb.org/miRDB/), miRanda (http://www.microrna.org/microrna/home.do), and miRWalk (http://www.ma.uni-heidelberg.de/apps/zmf/mirwalk/). Pathway analyses of miR-16 target genes were conducted using the KEGG pathway program (http://www.genome.jp/kegg/kegg2.html).

### mRNA quantitative assay

For the mRNA quantitative assay, cDNAs were synthesized from 1 μg of total RNA with random hexamers primers using Reverse Transcriptase (Applied Biosystems Inc.), and RT real-time PCR with SYBR Green (Applied Biosystems Inc.) was performed with the paired primers listed in Supplementary Table S1.

### Statistical analysis

All data were statistically analyzed using JMP software version 10.0 (SAS Institute Inc., Cary, NC, USA). The continuous variables are represented as mean ± standard deviation (SD) values, and the dichotomous variables are represented as number and percentage values. A T-test was used to analyze continuous variables. A Chi-square test was used where applicable for the univariate statistical analysis, and a Cox regression hazard model was used for multivariate analyses of OS and DFS. The survival plot was calculated by the Kaplan-Meier method, and the differences in survival rates were analyzed by the log-rank test. An analysis of covariance was performed to compare the mean levels of miR expression between subjects with different blood sugar levels. A two-tailed *P* value of less than 0.05 was considered statistically significant.

## SUPPLEMENTARY FIGURES AND TABLE


