# Supplementary material for: High blood sugar levels significantly impact the prognosis of colorectal cancer patients through down-regulation of microRNA-16 by targeting Myb and VEGFR2
Source: Oncotarget. 2016 Feb 25;7(14):18837–50. doi: 10.18632/oncotarget.7719 (PMC4951333; doi:10.18632/oncotarget.7719)
Supplement: Supplementary file 1 [file oncotarget-07-18837-s001.pdf]

SUPPLEMENTARY FIGURES AND TABLE

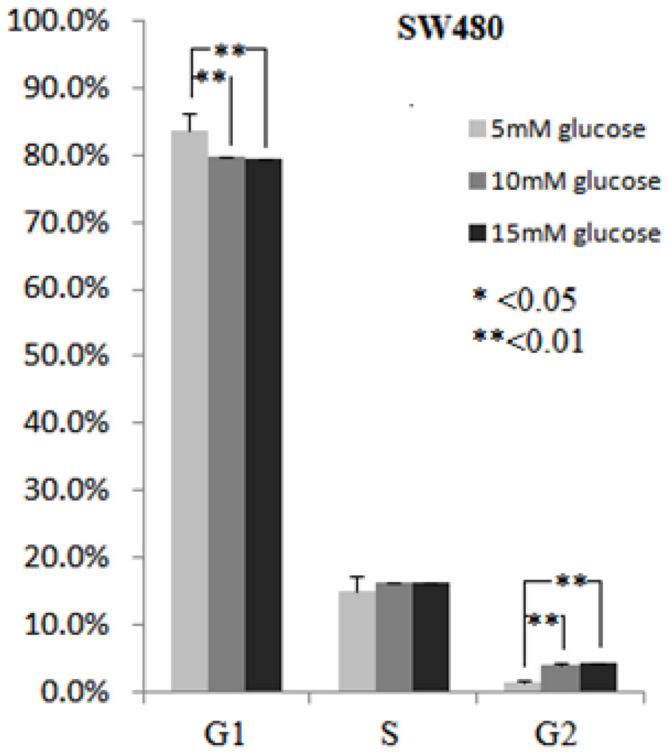

Supplementary Figure S1: SW480 colon cancer cell lines incubated in varying glucose conditions affect cell cycle. For SW480 cells after 48 h incubation, the accumulation in G1 population decreased significantly and the accumulation in G2 population increased significantly. The accumulation in G1 population was 83.57% in 5 mM of D-(+)-glucose *vs.* 79.63% in 10 mM of D-(+)-glucose and 79.35% in 15 mM of D-(+)-glucose (P=0.0079 and 0.0054, respectively), and the accumulation in G2 population was 1.52% in 5 mM of D-(+)-glucose *vs*. 4.19% in 10 mM of D-(+)-glucose and 4.36% in 15 mM of D-(+)-glucose (both P<0.0001).

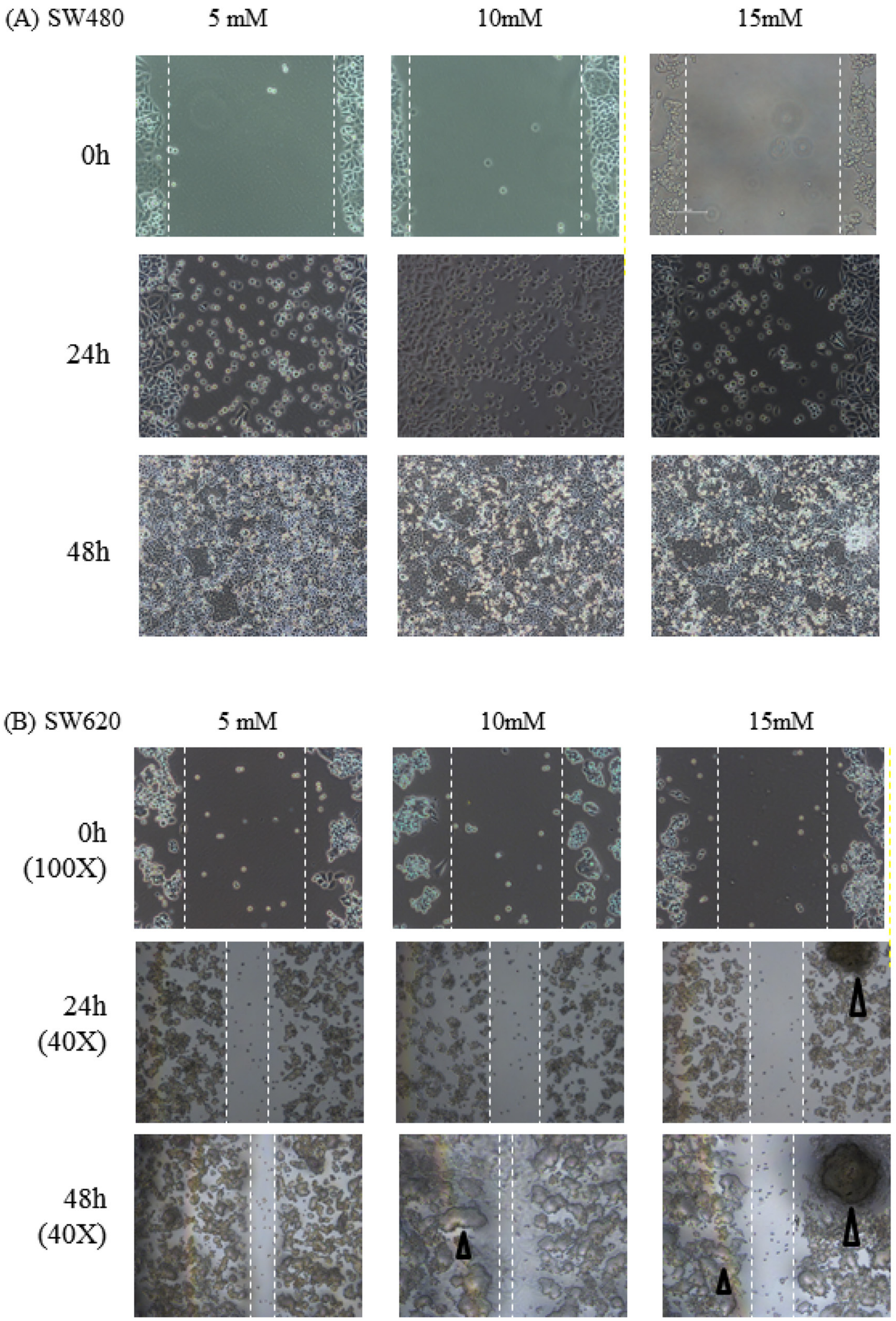

Supplementary Figure S2: SW480 and SW620 colon cancer cell lines were not suitable cell types for studying cell migration ability in the wound healing assay. A. SW480 cell migration ability can be detected by the wounding healing assay due to the SW480 cells' tendency to suspend from the crowded area and continue growing in the suspension medium. **B.** The wounding healing assay is not suitable for determining SW620 cell migration ability due to the SW620 cells' tendency to pack together in the crowded area and continue growing into lumps (indicated by arrows).

Supplementary Table S1: Primer sequences for the mRNA quantitative assay

Gene Name	Forward primer	Reverse primer
GADPH	GCAAATTCCATGGCACCGTC	TCGCCCCACTTGATTTTGG
Myb	GACAGCAGGTGCTACCAACA	CCATGAGGTCTGGTGTGGTC
Myc	CAGCTGCTTAGACGCTGGATT	GTAGAAATACGGCTGCACCGA
Raf1	CCGAGAGTCTTAATCGCGGG	AAAAAGGCAGCAGAAAGCCG
VEGFR2	CACCACTCAAACGCTGACATGTA	CCAACTGCCAATACCAGTGGA
